# Recent advances in the understanding and management of cystic fibrosis pulmonary exacerbations

**DOI:** 10.12688/f1000research.13926.1

**Published:** 2018-05-14

**Authors:** Kate Skolnik, Bradley S. Quon

**Affiliations:** 1 Division of Respirology, Department of Medicine, University of Calgary, Calgary, AB, Canada; 2 Centre for Heart Lung Innovation, St Paul’s Hospital, Department of Medicine, University of British Columbia, Vancouver, BC, Canada

**Keywords:** cystic fibrosis, exacerbations, treatment

## Abstract

Pulmonary exacerbations are common events in cystic fibrosis and have a profound impact on quality of life, morbidity, and mortality. Pulmonary exacerbation outcomes remain poor and a significant proportion of patients fail to recover their baseline lung function despite receiving aggressive treatment with intravenous antibiotics. This focused review provides an update on some of the recent advances that have taken place in our understanding of the epidemiology, pathophysiology, diagnosis, and management of pulmonary exacerbations in cystic fibrosis as well as direction for future study.

## Introduction

Despite improvements in lung function and nutritional outcomes for individuals with cystic fibrosis (CF) over the past decade, pulmonary exacerbation (PEx) remains common. In 2016, according to US Cystic Fibrosis Foundation Patient Registry data, one in three patients required at least one course of intravenous (IV) antibiotics to treat a PEx ^1^. Exacerbations have a profound impact on the morbidity and quality of life of individuals with CF, and unfortunately PEx outcomes remain suboptimal with poor recovery of baseline lung function following PEx treatment ^2^. As a result, efforts are under way within the CF research community to improve the management of these clinically impactful events. The focus of this review is to summarize some of the recent advances that have taken place in our understanding of the epidemiology, pathophysiology, diagnosis, and management of PEx in CF and to provide direction for future study.

## Pulmonary exacerbation definition and diagnosis

Although there is general agreement on the importance of PEx, there is no consensus definition of what constitutes a PEx by CF clinicians, researchers, and the broader CF community. However, an ideal or consensus PEx definition (or scoring system) is likely to remain elusive without a gold standard to compare it against. Varying PEx definitions employed in the CF literature have been a major impediment to research progress and have inherently confounded many of the studies included in this review. This caveat must be kept in mind while reading this review because of the potential lack of specificity about what is being studied and discussed.

In an attempt to move toward a consensus PEx definition, the EuroCFCare Working group has recommended the use of a modified Fuchs criteria to define a PEx which includes the need for additional antibiotic treatment (oral or IV) and a recent change of at least two of the following six criteria: change in sputum volume or color; increased cough; increased fatigue, malaise, or lethargy; anorexia or weight loss; decrease in pulmonary function by 10% or more or radiographic changes; and increased dyspnea ^3^. However, this definition has been criticized by some experts in the field because the diagnosis of PEx should be independent of the physician’s decision to treat and pulmonary function testing limits the age range of patients assessed. Some recent pediatric trials have used the PEx definition employed in the Early *Pseudomonas* Infection Control (EPIC) trial, which consists of one major criterion (decrease in forced expiratory volume in one second [FEV _1_] at least 10% from baseline with the previous 6 months; oxygen saturation less than 90% on room air or at least 5% decline from baseline; new lobar infiltrates or atelectasis on chest X-ray; hemoptysis) or two minor symptoms/signs (increased respiratory rate; new or increased adventitial sounds on lung exam; weight loss of at least 5% in the previous 6 months; increased cough; decreased exercise tolerance; increased chest congestion or change in sputum) for at least 5 days or with significant symptom severity ^4^.

Despite the lack of a consensus PEx definition, recent studies have focused on strategies to diagnose CF PEx earlier given the risk of poor outcomes. In the Standardized Treatment of Pulmonary Exacerbations (STOP) study, a multi-site observational study of patients with CF treated with IV antibiotics in hospital, most patients (85%) described symptoms more than 7 days before admission and nearly one-third (32%) had symptoms more than 21 days beforehand ^5^. Less than half (48%) of these individuals received oral antibiotics prior to admission, suggesting that certain patients may have prolonged symptoms in keeping with PEx but, due to delays in diagnosis, are not started on treatment. For these reasons, there has been a recent focus on home monitoring of symptoms and lung function to promote earlier PEx detection and treatment to prevent irreversible lung damage.

A multi-center study from the Netherlands examining electronic home monitoring of symptoms and lung function for early PEx detection confirmed a change in symptoms at least 4 weeks prior to PEx diagnosis in most patients, and further symptom deterioration in the 2 weeks prior to PEx diagnosis ^6^. However, a large multi-center randomized trial recently conducted in the US demonstrated that although electronic home monitoring of symptoms and spirometry is feasible and leads to earlier and more frequent diagnoses of PEx compared with usual care, this strategy does not lead to less lung function lost over 1 year ^7^. An earlier diagnosis in the home-monitoring arm led to more frequent use of oral (versus IV) antibiotics compared with the usual-care arm (67% versus 43%), and this might have resulted in a higher rate of non-recovery of FEV _1_ % predicted to within 5% of baseline (47% versus 21%) due to a less robust response to oral antibiotic treatment ^7^. The authors concluded that identifying PEx earlier may not be sufficient and that future studies must also find better approaches to treatment of exacerbations once they are detected. However, there may still be utility for home monitoring for early PEx detection, particularly for individuals who are poor perceivers of their symptoms, have frequent exacerbations, or reside in rural areas which may delay access to care.

## Pulmonary exacerbation epidemiology

### Pulmonary exacerbation prevalence

The proportion of patients requiring at least one course of IV antibiotics per year has not decreased significantly over the past decade ^1^. Although this might seem concerning in light of the overall improvements in lung function observed over the same time period, this appears to be driven by a lower threshold among clinicians to diagnose and treat PEx ^8^. Some important studies have highlighted the wide variability in the recognition and treatment of PEx between CF clinics and individual CF physicians in both the US and the UK ^9, 10^. A multi-center study in the UK demonstrated higher IV antibiotic use among centers with higher baseline FEV _1_ % predicted ^10^, whereas an older multi-center US study found variability among centers with respect to care (those with a higher median FEV _1_ % predicted had more frequent monitoring and antibiotics) ^9^. These findings have led to quality improvement initiatives to reduce variability ^11, 12^.

### Pulmonary exacerbation risk factors

In order to prevent PEx and their associated sequelae, there has been a great deal of interest in identifying the risk factors for future PEx. VanDevanter *et al*. investigated factors associated with increased risk of PEx requiring IV antibiotics ^13^. The study found that, out of numerous clinical variables, including sputum microbiology and treatment characteristics, the strongest risk factor for a PEx requiring IV antibiotic therapy was the occurrence of a PEx requiring IV antibiotics in the preceding year ^13^. Not surprisingly, individuals with three or more exacerbations had the highest risk of future PEx compared with those with one or two exacerbations ^13^.

### Pulmonary exacerbation outcomes

With regard to PEx treatment outcomes, exacerbations treated with oral antibiotics are often considered to be milder events, whereas those treated with IV antibiotics are considered to represent more severe events. However, labeling a PEx on the basis of route of antibiotic treatment (oral versus IV) is limited and potentially biased, since factors other than PEx severity (based on symptoms, inflammatory markers, or lung function decline or a combination of these) may influence the decision of antibiotic route. For instance, IV antibiotics may be chosen on the basis of medication allergies/intolerances, bacterial resistance to oral antibiotics, or other non-disease severity-related factors (such as psychosocial issues or insurance coverage).

In support of the concept that exacerbations treated with oral antibiotics may not represent mild events, based on a recent retrospective study using the Toronto CF database from 2000 to 2014, nearly 20% of exacerbations treated with oral antibiotics did not recover to within 90% of baseline FEV _1_ % predicted within 3 months of treatment ^14^. Furthermore, the greater the number of cumulative oral antibiotic-treated events over the study period, the steeper the rate of lung function decline ^14^. Consequently, close follow-up post-treatment is warranted to ensure recovery, and patients with repeated exacerbations treated with oral antibiotics may warrant more intensive treatment with IV antibiotics.

Even among patients who receive aggressive treatment with IV antibiotics, PEx outcomes remain suboptimal. Based on CF registry data from both the US and Canada, 25% of individuals who experience exacerbations fail to recover baseline lung function as defined by 90% of baseline within 3 months of treatment ^2, 15^. Factors independently associated with non-response included female sex; malnourishment; pancreatic insufficiency; persistent infection with *Pseudomonas aeruginosa*, *Burkholderia cepacia* complex, or methicillin-resistant *Staphylococcus aureus*; allergic bronchopulmonary aspergillosis; larger drop in FEV _1_ % predicted at the time of PEx; and longer time from baseline spirometric assessment ^2^.

It is important to recognize that the proportion of non-responders can vary substantially depending on the definition of “response” used. For example, when a definition of FEV _1_ improvement to 90% of baseline is employed, 25% of exacerbations are classified as non-responders, whereas an improvement to 100% of baseline yields a non-response rate as high as 60% ^16^. Furthermore, it should be noted that up to 25% of patients in the STOP trial had their best lung function at the time of PEx diagnosis and therefore a sizeable proportion of patients will be defined as responders even before treatment has started, leading to underestimation of treatment non-response. Most studies have used the best FEV _1_ in the 3 months following the end of IV antibiotic treatment as the follow-up FEV _1_, since lung function improvement can continue following the completion of IV antibiotics and therefore end-of-treatment values can underestimate rates of response.

## Pulmonary exacerbation triggers

Viral respiratory tract infections have been estimated to be associated with about 50% of exacerbations and this might explain why exacerbations are more frequent during the winter months ^17– 19^. The most common viral pathogens include rhinovirus, respiratory syncytial virus (RSV) (in children), parainfluenza, influenza, adenovirus, coronavirus, and coxsackie/echovirus ^17, 18, 20, 21^. It was previously hypothesized that viruses could increase bacterial density of chronic colonizing organisms; however, a recent study has refuted this, demonstrating no change in *P. aeruginosa* density between viral- versus non-viral-associated PEx ^17^. Interestingly, anti-viral interferon signaling in response to RSV infection is capable of inducing *P. aeruginosa* biofilm formation through dysregulated iron homeostasis and this could represent a putative mechanism for viral-triggered PEx but warrants further study ^22^. It is also important to note that although there appears to be a strong association between viruses and PEx, this does not necessarily imply a causal relationship, as studies have demonstrated the presence of virus even when patients are well (suggesting asymptomatic nasopharyngeal carriage) ^23, 24^.

While non-viral-associated exacerbations were also believed to be the result of increased bacterial density of the chronic primary pathogen, recent studies have challenged this dogma. Multiple studies have demonstrated no significant changes in bacterial density from stable to PEx state ^25– 28^. Although decreases in bacterial density are observed following anti-microbial treatment, these effects are transient and are poorly predictive of clinical response ^28– 30^. Research has shown that CF airway infections are polymicrobial and that interactions between these microbes may increase or decrease pathogenicity ^31^. It is believed by some experts that organisms (such as anaerobes) can interact with the primary pathogen to enhance virulence without a change in density ^31^. For example, a recent study evaluating airway bacterial communities with 16S rRNA sequencing found no significant differences in bacterial community diversity or density between paired stable and PEx samples, but there was a change in community structure for a subgroup of patients ^26^. Furthermore, the absolute and relative abundance of *Gemella spp*. increased in the majority of samples from stable to PEx state, and this was most discriminative of health status (stable versus PEx) ^26^.

Air pollution is also an important trigger of PEx, and a seminal study linking the US CF National Registry to the US Environmental Protection Agency Aerometric Information Retrieval System demonstrated a significant association between annual average exposure to particulate matter and risk of PEx ^32^. A recent case-crossover analysis confirmed this finding and found that increased exposure to particulate matter less than 10 mm in diameter (PM _10_), nitrogen dioxide, and ozone was associated with increased need for oral or IV antibiotics for PEx on the day of exposure ^33^.

## Pulmonary exacerbation treatment

### Treatment endpoints

In general, the goal of PEx treatment is to improve symptoms and recover lost lung function ^34^. Based on the STOP study, physicians identified recovery of lung function as the primary objective of treatment in 53% of exacerbations compared with improvement of symptoms in 43% of exacerbations ^5^. There is considerable variability as to what constitutes an acceptable threshold for FEV _1_ improvement before antibiotics can be stopped. Based on the STOP study, the mean FEV _1_ improvement was 9% (standard deviation [SD] 10%) predicted at the end of IV antibiotic treatment and 7% (SD 11%) predicted at day 28. Patients with baseline FEV _1_ of more than 50% predicted had a greater increase in FEV _1_ % predicted from admission to day 28 than patients with baseline FEV _1_ less than 50% predicted (10% versus 3%). Interestingly, there was discordance between physician treatment targets in terms of lung function improvement and evaluation of treatment success. Based on the STOP study, 84% of clinicians deemed PEx treatment successful, although only 61% of patients achieved at least 90% of their target FEV _1_ by the end of IV antibiotic therapy ^35^.

The treatment endpoint for an individual patient is likely to depend on the primary motivating factor for treatment. In the STOP study, a significant proportion of patients (20%) were admitted for IV antibiotics despite presenting with their best recorded FEV _1_ % predicted in the prior 6 months ^5^. In these cases, symptoms were the primary driver for treatment and symptom resolution would be the most appropriate endpoint of treatment response. In general, exacerbations are more likely to be defined based on symptoms alone in children compared with adults (in whom drop in lung function may be more likely to occur) ^36^. The most widely used daily scoring system for monitoring of respiratory/infectious symptoms during PEx treatment is the Cystic Fibrosis Respiratory Symptom Diary – Chronic Respiratory Infection Symptom Score (CFRSD-CRISS) ^37^. Total scores range from 0 to 100, and an 11-point decrease is considered clinically significant ^38^. Based on STOP, CFRSD-CRISS decreased by 26.1 (95% confidence interval 23.8–28.3) and 83% of patients achieved a clinically significant improvement ^35^. However, it should be noted that the CFRSD-CRISS has limitations in that it has been used and evaluated in a research setting only and has not been accepted by the US Food and Drug Administration as a validated endpoint.

Whereas short-term goals of PEx treatment are to recover lost lung function and improve symptoms, long-term treatment goals generally are to prevent recurrent events and reduce the rate of lung function decline. Interestingly, a recent study found that symptom improvement (that is, CFRSD-CRISS score) in response to PEx treatment is poorly predictive of long-term response in terms of recovery of baseline lung function at 3 months or time to next IV antibiotics; however, immediate FEV _1_ response (>10% relative improvement) was predictive of recovery of baseline FEV _1_ by 3 months ^39^. Synthesizing all of this evidence suggests that a composite outcome that includes symptom and lung function improvement might be the most appropriate endpoint in prospective studies examining PEx therapies.

### Treatment controversies

**Figure 1.**
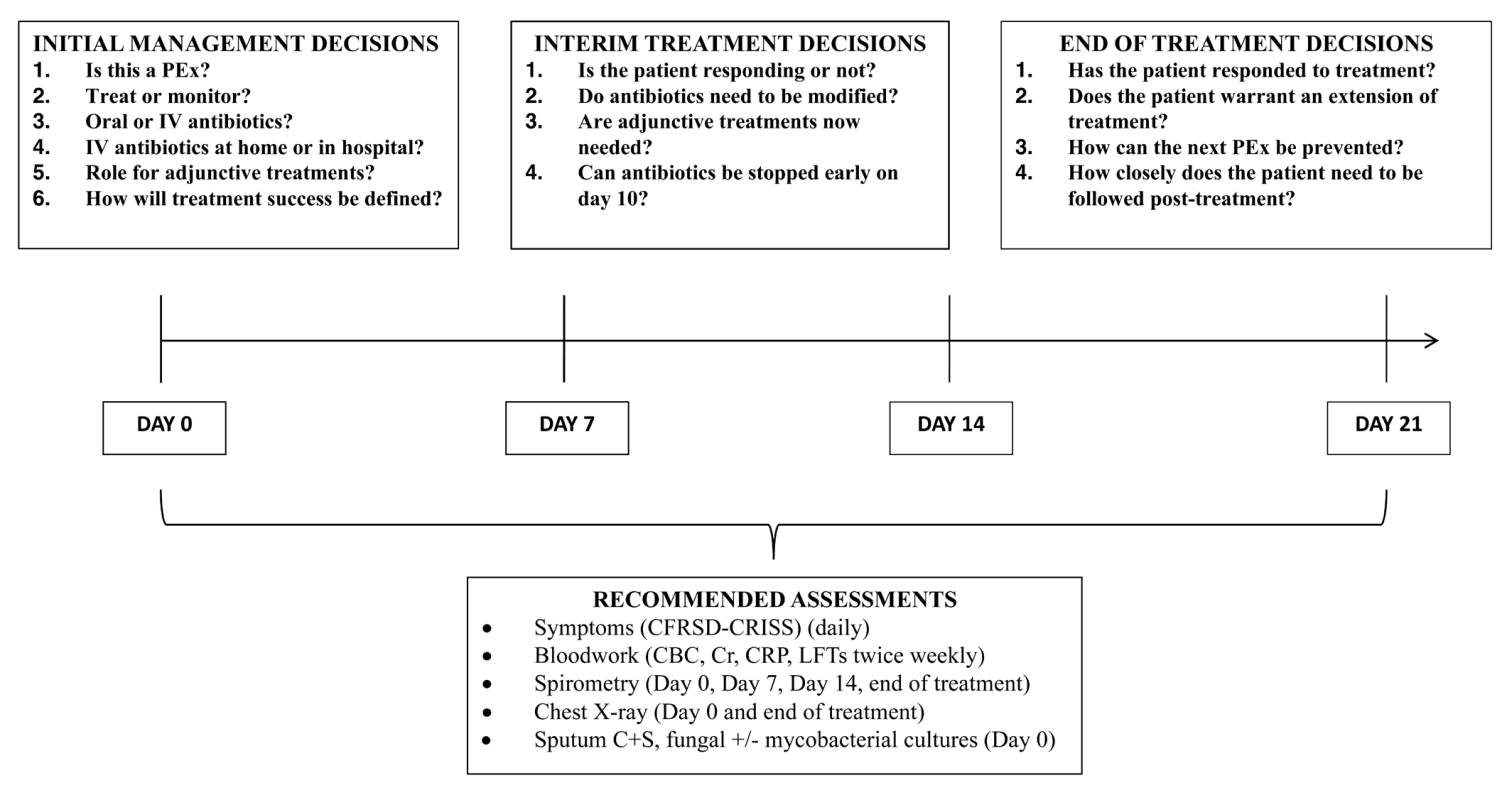
Outline of pulmonary exacerbation treatment decisions. CBC, complete blood count; CFRSD-CRISS, Cystic Fibrosis Respiratory Symptom Diary-Chronic Respiratory Infection Symptom Score; Cr, creatinine; CRP, C-reactive protein; C+S, culture and sensitivity; IV, intravenous; LFT, liver function test; PEx, pulmonary exacerbation.

Based on the 2009 Cystic Fibrosis Foundation PEx treatment guidelines, there was insufficient evidence to provide recommendations on a number of decisions related to PEx management, including site of treatment (inpatient versus outpatient), antibiotic duration, and the use of adjunctive therapies (such as systemic steroids) ( Figure 1) ^40^. Since this publication, observational studies have provided additional insights into these treatment controversies and randomized controlled trials (RCTs) are ongoing or have been recently completed.

### Site of treatment

There are some circumstances in which admission to hospital for optimal PEx treatment is clearly indicated (such as hypoxia, complications such as pneumothorax, or co-morbidities such as renal failure), but in many situations outpatient treatment with IV antibiotics appears to be a reasonable option. However, in deciding on the optimal treatment setting, an important factor is not just safety and feasibility but also whether one treatment setting is more efficacious than the other. One small randomized, two-factor, mixed-design comparative study involving 17 adults with CF has been performed and showed similar results for most outcome measures for home and hospital treatment ^41^. Owing to selection bias, the results of a larger RCT of the two treatment settings may not be generalizable and will be challenging to interpret because of lack of blinding, attrition bias, high rates of crossover, and lack of standardized resources for home care at various care centers ^40^.

A small, single-center retrospective study examined 143 PEx events from 50 patients and compared hospital with home IV therapy ^42^. The baseline characteristics between the two groups were similar; however, the hospitalized group had a greater improvement in lung function with a shorter duration of therapy ^42^. In a recent observational study of pediatric CF patients from the Epidemiologic Study of CF (ESCF), those with an acute decline in lung function were more likely to recover to within 90% of baseline FEV _1_ when receiving treatment as an inpatient versus outpatient ^43^. However, observational studies involving treatment are often confounded by indication bias. In other words, patients are not randomly assigned and therefore the intensity of treatments is not standardized between comparisons; ultimately, patients in hospital might receive more intensive treatments. Another recent study that also used the ESCF employed statistical approaches to control for indication bias and found that PEx with a greater proportion of days treated as an inpatient (versus outpatient) with IV antibiotics was more likely to lead to return of FEV _1_ % predicted to at least 90% of baseline ^44^. Although this observational study is not definitive (as there was likely residual confounding), the benefits of inpatient PEx treatment are likely due to multiple factors other than the IV antibiotics alone, including improved medication adherence, better nutrition, real-time adjustment of adjunct therapies, increased airway clearance treatments, and increased rest.

Ultimately, the decision regarding treatment site should be made based on careful consideration of patient factors (with a low threshold for inpatient treatment if there are concerns about patient reliability and adherence to airway clearance therapies, sufficient home supports, or more complex IV antibiotic regimens) ^40^.

### Duration of antibiotics

For historical reasons, CF exacerbations are typically treated for 14 days. Based on the STOP study, the mean duration of treatment was 15.9 (SD 6.0) days; however, 11% of patients received treatment for 10 days or less and 60% received treatment for more than 14 days with no significant differences in duration for individuals younger than 18 years old versus those older than 18 years old ^35^. There have been no randomized studies examining the duration of IV antibiotics to provide evidence-based recommendations. An observational study that used the US CF Foundation Patient Registry found that FEV _1_ % predicted plateaus by day 10 of treatment and duration of treatment did not influence time until next PEx ^45^. More recent retrospective data on the benefits of prolonging antibiotic treatment are conflicting, as a study using the Toronto CF Database found improved outcomes with longer treatment duration (>14 days) whereas a larger study using data from the ESCF did not find a significant association between treatment duration and rate of recovery of FEV _1_ % predicted to within 90% of baseline ^16, 44^. However, as in observational studies of treatment setting, interpretation is limited by indication bias, since patient factors influence treatment duration (patients with lower lung function received longer treatment courses) ^45^. A large multi-center study (STOP2) is under way in the US examining IV antibiotic duration since it was identified as the most important research question by CF physicians and patients/caregivers. A divergent trial design is being used and randomly assigns patients to 10 versus 14 days or 14 versus 21 days of IV antibiotics depending on initial symptom and lung function response by day 7 ^46^.

### Adjunctive therapies

A recent study evaluating inpatient PEx treatment practices for pediatric CF patients across the US demonstrated wide variability in the use of adjunctive treatments, including hypertonic saline, azithromycin, and systemic corticosteroids ^47^. Several adjunct therapies for the treatment of CF PEx have recently been evaluated or are currently under investigation. These adjunct therapies function by either optimizing airway clearance or reducing airway inflammation.

Although nebulized hypertonic saline is well established as a strategy for PEx prevention ^48^, it has only recently been studied as an adjunct during PEx treatment. In an RCT, Dentice *et al*. compared PEx outcomes in individuals randomly assigned to nebulized 7% hypertonic saline versus taste-masked control thrice daily ^49^. The majority of patients in both groups had either never or only intermittently used hypertonic saline prior to enrollment. Although this study did not meet its primary endpoint in terms of reduced length of hospital stay, there was greater improvement in symptoms and higher rates of FEV _1_ recovery in the hypertonic saline group ^49^. Furthermore, the study provided reassurance that nebulized hypertonic saline was safe to start (or increase) in the context of acute PEx ^49^.

Another recent study examined doxycycline as an adjunct therapy for CF PEx, acting as a small-molecule inhibitor of matrix metalloproteinase-9 (MMP-9), which has been implicated in CF airway pathophysiology, particularly during PEx ^50, 51^. This single-center RCT randomly assigned 39 CF patients with PEx requiring inpatient care to either doxycycline 100 mg orally twice daily or placebo for 8 days in addition to standard patient care (IV antibiotics and increased airway clearance) ^52^. Compared with the placebo group, the doxycycline group had a significant decrease in total and active sputum MMP-9 levels, improved protease-antiprotease imbalance in the airways, greater improvement in FEV _1_ % predicted from admission, and longer time to next PEx ^52^. Whether doxycycline has direct effects on dysregulated protease activity versus indirect effects due to changes in the microbiome remains unclear, but this single-center study has set the stage for a larger multi-center placebo-controlled RCT ^52^.

Although systemic corticosteroids are used in up to 20% of CF exacerbations ^35, 39^, there is limited evidence to support their use ^40^. Just one small pilot placebo-controlled study involving 24 patients (≥10 years old) examined oral prednisone (2 mg/kg per day up to a maximum of 60 mg divided twice daily) versus placebo for the first 5 days as an adjunct to standard-of-care PEx treatment ^53^. Although there was no significant effect on lung function, symptom improvement, or sputum inflammatory markers compared with placebo, the study was underpowered to evaluate treatment effects ^53^. A multi-center, randomized, placebo-controlled trial evaluating prednisone—referred to as the Prednisone in CF Pulmonary Exacerbation (PIPE) study—is under way and involves six pediatric and adult CF clinics across Canada. This study will provide more definitive evidence regarding the role of systemic corticosteroids during PEx. Patients receiving IV antibiotic treatment and who have not responded to standard of care alone by day 7 (that is, not recovered at least 90% of their baseline FEV _1_ % predicted) will be randomly assigned to prednisone (2 mg/kg per day up to a maximum of 60 mg divided twice daily) or placebo for 7 days.

## Pulmonary exacerbation biomarkers

In the field of CF, there is tremendous interest in identifying a biomarker that could aid in the earlier diagnosis of a CF PEx or assist in tracking the response to PEx treatment or do both. Earlier diagnosis can allow for the timelier initiation of treatment, which might result in better PEx outcomes. A reliable biomarker of response to treatment could also identify non-responders earlier during the course of treatment so that therapies can be modified or extended accordingly.

The majority of biomarkers in relation to PEx have focused on inflammation in the sputum and blood ^54, 55^. Although sputum is an attractive option because it most closely reflects airway inflammation, its evaluation has been limited to research studies because it can be challenging to collect and process in clinical laboratories. Several sputum biomarkers of inflammation, including interleukin-8 (IL-8), neutrophil elastase (NE), calprotectin, club cell secretory protein (CCSP), and MMP-9, have been investigated during PEx ^56– 59^. Although most of these sputum biomarkers change significantly from stable to PEx state or following PEx treatment, the results have been variable between studies. Sputum NE appears to be promising, as a recent study by Waters *et al*. found that a decrease in sputum NE was independently associated with response to IV antibiotic treatment at day 14 and higher NE levels at day 14 were associated with greater risk of subsequent PEx ^57^.

Among blood biomarkers, numerous markers of inflammation have been evaluated as recently reviewed ^60^. Serum C-reactive protein (CRP) and calprotectin have been the most extensively studied in the context of PEx and offer the most promise for clinical use ^54^. All studies evaluating CRP and calprotectin in CF have demonstrated significant reductions in levels from beginning to end of PEx treatment ^54, 56, 58^. However, a recent study demonstrated that CRP increases in 25% of patients during the first 5 days of IV antibiotic treatment prior to decreasing, thus making it a challenging biomarker to monitor early response to treatment ^61^. Individuals with persistently elevated CRP and calprotectin levels following IV antibiotics also experience a shorter time to re-exacerbation ^57, 62^.

Although several candidate biomarkers that correlate with clinical outcomes during PEx treatment have been identified, it remains unclear whether their prospective use can influence treatment decisions to improve PEx outcomes and whether they add incremental utility to monitoring of symptoms and lung function alone.

## Future directions

Although small but incremental progress is being made in our understanding of PEx in CF on the basis of observational studies, there remain several gaps in knowledge and a need for more interventional studies to guide evidence-based practice. The CF community is eagerly awaiting the results of the aforementioned RCTs evaluating various IV antibiotic treatment durations and the adjunctive use of systemic corticosteroids and doxycycline, as these have the potential to improve PEx outcomes. In addition, a greater understanding of the sequence of events leading to a PEx at a molecular level is required to improve PEx phenotyping and to guide the development of more targeted treatments.
